# Abdominal Radiotherapy: A Major Determinant of Metabolic Syndrome in Nephroblastoma and Neuroblastoma Survivors

**DOI:** 10.1371/journal.pone.0052237

**Published:** 2012-12-14

**Authors:** Marjolein van Waas, Sebastian J. C. M. M. Neggers, Hein Raat, Caroline M. van Rij, Rob Pieters, Marry M. van den Heuvel-Eibrink

**Affiliations:** 1 Department of Pediatric Oncology/Hematology, Erasmus MC - Sophia Children’s Hospital Rotterdam, Rotterdam, The Netherlands; 2 Department of Medicine, Section Endocrinology, Erasmus University Medical Center Rotterdam, Rotterdam, The Netherlands; 3 Department of Public Health, Erasmus University Medical Centre Rotterdam, Rotterdam, The Netherlands; 4 Department of Radiation Oncology, Erasmus University Medical Center Rotterdam, Rotterdam, The Netherlands; University of Leicester, United Kingdom

## Abstract

**Background:**

Reports on metabolic syndrome in nephroblastoma and neuroblastoma survivors are scarce. Aim was to evaluate the occurrence of and the contribution of treatment regimens to the metabolic syndrome.

**Patients and Methods:**

In this prospective study 164 subjects participated (67 adult long-term nephroblastoma survivors (28 females), 36 adult long-term neuroblastoma survivors (21 females) and 61 control subjects (28 females)). Controls were recruited cross-sectionally. Waist and hip circumference as well as blood pressure were measured. Body composition and abdominal fat were assessed by dual energy X-ray absorptiometry (DXA-scan). Laboratory measurements included fasting triglyceride, high density lipoprotein-cholesterol (HDL-C), glucose, insulin, low-density lipoprotein-cholesterol (LDL-C) and free fatty acids (FFA) levels.

**Results:**

Median age at follow-up was 30 (range 19–51) years in survivors and 32 (range 18–62) years in controls. Median follow-up time in survivors was 26 (6–49) years. Nephroblastoma (OR = 5.2, P<0.0001) and neuroblastoma (OR 6.5, P<0.001) survivors had more components of the metabolic syndrome than controls. Survivors treated with abdominal irradiation had higher blood pressure, triglycerides, LDL-C, FFA and lower waist circumference. The latter can not be regarded as a reliable factor in these survivors as radiation affects the waist circumference. When total fat percentage was used as a surrogate marker of adiposity the metabolic syndrome was three times more frequent in abdominally irradiated survivors (27.5%) than in non-irradiated survivors (9.1%, P = 0.018).

**Conclusions:**

Nephroblastoma and neuroblastoma survivors are at increased risk for developing components of metabolic syndrome, especially after abdominal irradiation. We emphasize that survivors treated with abdominal irradiation need alternative adiposity measurements for assessment of metabolic syndrome.

## Introduction

Survival of childhood cancer has increased significantly over the past few decades, leading to increased recognition and knowledge of late effects. As treatment of childhood cancer is administered in a growing and developing individual, organs and tissues might be affected in a different and more severe way than in adults. The mean five-year survival for nephroblastoma and neuroblastoma survivors is 92% and 55%, respectively [1], [2]. Although endocrine late sequelae of pediatric cancer treatment are reported [3], [4], [5], [6], [7], studies in nephroblastoma and neuroblastoma survivors are scarce. Chemotherapy including anthracyclines, actinomycin and cyclophosphamide are administered in a considerable subset of these survivors. Moreover, abdominal radiotherapy is administered which includes the internal organs in the radiation field. In addition, late effects after treatment in these survivors may be aggravated by the removal of the affected organ. Subsequently, development of the metabolic syndrome may occur. The Childhood Cancer Survivor Study found exposure to total body irradiation or abdominal plus chest irradiation to be associated with components of the metabolic syndrome such as dyslipidemia, arterial hypertension and diabetes [8]. In addition, increased prevalence of hypertension after abdominal irradiation was observed in a study including 62 nephroblastoma survivors [9] and increased prevalence of cardiovascular events after abdominal irradiation was reported in a study including 185 nephroblastoma survivors [10].

Metabolic syndrome is known to increase the risk for diabetes and cardiovascular diseases [11], [12]. Adipose tissue, or more specifically visceral fat, which is located around the internal organs, plays a central role in the pathophysiology of metabolic syndrome. Adipose tissue has been recognized as a highly active metabolic organ involved in the production of several hormones [13]. Excess of visceral fat is strongly correlated with cardiovascular diseases, type 2 diabetes, insulin resistance and inflammatory diseases [14], [15], [16], [17]. The easiest way to measure visceral fat is by measuring waist circumference. However, survivors of specific cancer types such as nephroblastoma and neuroblastoma suffer from scarred abdominal areas as a result from surgery and truncal soft tissue hypoplasia caused by abdominal irradiation [18], [19], [20]. It is conceivable that these anatomical derangements induce incorrect interpretation of abdominal adiposity. Subsequently, although this issue has never been raised in childhood cancer survivor studies, underscoring of the frequency of the metabolic syndrome may occur.

Metabolic syndrome is defined for use in a clinical setting, however other metabolic parameters such as measures of insulin resistance and lipids provide additional information about cardiovascular risk. Our primary objective was to evaluate the frequency of (the components of) metabolic syndrome and associated measures of insulin resistance and dyslipidemia in adult long-term childhood nephroblastoma and neuroblastoma survivors in comparison with a control group. Secondary, we aimed to determine the influence of radiotherapy, chemotherapy and surgery on the occurrence of metabolic syndrome.

## Methods

### Patients

All long-term (≥5 years after cessation of treatment) adult survivors of childhood nephroblastoma and neuroblastoma, treated between 1961–2004 in the Erasmus MC-Sophia Children’s Hospital that regularly visit the Late Effects outpatient clinic were invited to participate in this prospective study. Written informed consent was obtained according to the Helsinki declaration and the study was approved by the local medical ethical committee of the Erasmus Medical Centre, Rotterdam, the Netherlands (NTR 2814). A controlgroup, consisting of siblings, friends or neighbours, preferably of the same sex and within an age range of five years of the survivor, was cross-sectionally recruited.

### Methods

**Figure 1:**
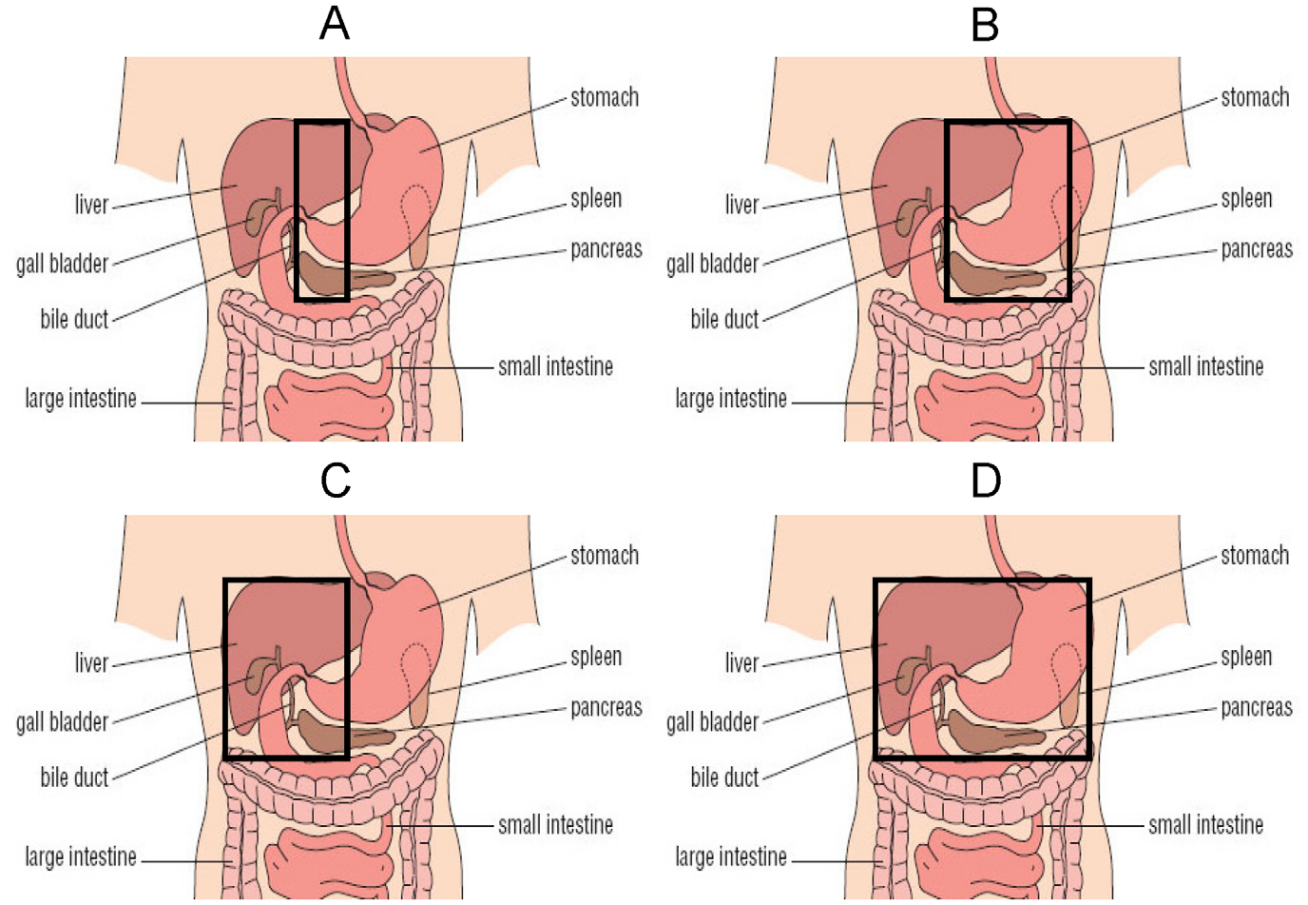
The black box indicates a schematic interpretation of the radiation field. A Part of pancreas (i.e. head and part of tail) and part of liver in radiation field **B** Total pancreas (i.e. head and tail) and part of liver in radiation field **C** Part of pancreas (i.e. head and part of tail) and total liver in radiation field **D** Total pancreas (i.e. head and tail) and total liver in radiation field **Categories: B+D** Total pancreas in radiation field **A+C** Part of pancreas in radiation field **C+D** Total liver in radiation field **A+B** Part of liver in radiation field Original figure was retrieved from http://openlearn.open.ac.uk.

Disease and treatment data were obtained from our local database. Baseline data regarding weight, height and BMI at diagnosis were extracted from the medical records. Data regarding (partial) nephrectomy and (partial) unilateral adrenalectomy were confirmed from pathological reports. Abdominal irradiation was categorized according to location, i.e.: A) spine plus 1.5 cm on the right and on the left hemi-abdomen, B) left hemi-abdomen including the spine plus 1.5 cm on the right hemi-abdomen, C) right hemi-abdomen including the spine plus 1.5 cm on the left hemi-abdomen, D) total abdomen. Consequently, the following categories according to organs involved in the radiation field were created: Total pancreas (B+D), part of pancreas (A+C), total liver (C+D) and part of liver (A+B) (Figure 1). Information regarding smoking status, educational level, statin and antihypertensive medication and diabetes was collected using a questionnaire. Smoking status was defined as non-smoker, former smoker or current smoker. Educational level was defined by the highest level of educational attainment as selected from three categories based on the Dutch educational system. Daily life physical activity was assessed by the Short Questionnaire to Assess Health-enhancing physical activity (SQUASH) [21]. Baseline characteristics are shown in Table 1. Height was measured to the nearest millimeter using a Harpenden Stadiometer and weight was assessed wearing underwear only to the nearest 0.1 kg with a standard clinical balance. Body mass index (BMI) was calculated (weight(kg))/(height(cm)^2^) [22] and expressed in standard deviation scores (SDS) [23], [24]. Waist and hip circumference were measured to the nearest 1 cm, midway between last rib and the iliac crest and at the maximum circumference of the buttocks, respectively [25]. Waist-hip-ratio was calculated. Blood pressure was measured with the subject in sitting position after an hour of rest on the right arm with the Dinamap® Procare and was defined as the mean of three measurements. The metabolic syndrome is a constellation of insulin resistance, adiposity, hypertension and dyslipidemia. According to the revised National Cholesterol Education Program/Adult Treatment Panel III (NCEP/ATP III) criteria, participants with at least three of the following components were diagnosed with metabolic syndrome: Waist circumference (as a proxy for visceral fat) >102 cm in males or >88 cm in females; triglycerides ≥1.7 mmol/l or treatment for dyslipidemia; high density lipoprotein-cholesterol (HDL-C) <1.03 mmol/l in males or <1.30 mmol/l in females; fasting plasma glucose (as a measure of insulin resistance) ≥5.6 mmol/l or treatment for type 2 diabetes; blood pressure ≥130/85 mmHg or treatment for hypertension [26]. Data on total body fat mass (kg), lean body mass (kg) and percentage of body fat were retrieved from dual energy X-ray absorptiometry (DXA, GE Lunar Prodigy, USA), which was performed in survivors only. In addition, visceral fat percentage was calculated from intra-abdominal fat (kg) and total fat (kg) using the DXA scan [27]. Values for lean body mass and total fat percentage were compared with normal Dutch reference values and calculated as SDS [28].

**Table 1:** Baseline characteristics of study participants.

	Nephroblastoma		Neuroblastoma		Controls
**N**	67		36		61
**Male/female**	39/28		15/21		33/28
**Age at follow-up (yrs)**	30.2 (18.8–50.8)		29.6 (20.4–46.2)		31.8 (18.0–61.8)
**Age at diagnosis (yrs)**	3.3 (0.0–12.7)		0.8 (0.0–11.7)		n.a.
**Follow-up time** * **(yrs)**	26.2 (6.4–48.9)		27.8 (15.0–44.4)		n.a.
**BMI (SDS)** **	−0.52 (−1.21–0.13)		−0.58 (−1.99–0.16)		0.08 (−0.51–0.68)
**BMI at diagnosis (SDS)** **	0.08 (−0.63–0.80)		0.31 (−0.61–0.74)		n.a.
**Physical activity score** **	8280 (6660–12000)		7130 (5843–9005)		8330 (6570–11370)
**Non smoker**	66%		50%		53%
**Former smoker**	12%		19%		16%
**Smoker**	22%		31%		31%
**Low educational level**	21%		22%		16%
**Medium educational level**	36%		33%		48%
**High educational level**	43%		45%		36%
**(Partial) adrenalectomy (N)**	33/67		13/36		n.a.
**(Partial) nephrectomy (N)**	67/67		7/36		n.a.
**Radiotherapy abdomen (N)**	35/67		7/36		n.a.
**Radiotherapy thorax (N)**	0/67		2/36		n.a.
**Radiotherapy neck (N)**	0/67		1/36		n.a.
**Radiotherapy spine (N)**	0/67		1/36		n.a.
**Cumulative dose radiotherapy (gy)**	20 (15–40)		20 (10–30)		n.a.
**Chemotherapy (N)**	59/67		31/36		n.a.
	**N**	**TCD**	**N**	**TCD**	
**Vincristin (mg/m^2^)**	51	22.0 (6.0–93.0)	16	22.8 (5.0–65.0)	n.a.
**Actinomycin D (mg/m^2^)**	48	10.9 (0.1–24.8)	0	n.a.	n.a.
**Anthracyclines (mg/m^2^)**	18	250 (100–450)	12	210 (100–315)	n.a.
**Cyclophosphamide (mg/m^2^)**	2	3825 (250–7400)	29	7350 (3150–45990)	n.a.
**Cisplatin (mg/m^2^)**	0	0	6	450 (270–630)	n.a.
**Teniposide (mg/m^2^)**	0	0	6	500 (300–700)	n.a.
**Dacarbazine (mg/m^2^)**	2	14.7 (13.5–15.8)	0	0	n.a.
**Iphosphamide (mg/m^2^)**	2	33000 (30000–36000)	0	0	n.a.

Data expressed as median (range) unless specified otherwise.

* Time after cessation of treatment.

** Data expressed as median (interquartile range).

**n.a.** = not applicable, Gy = gray, TCD =  total cumulative dose.

### Laboratory Measurements

Fasting blood samples were obtained from an intravenous-cannula before 10∶00 a.m. Serum values of triglyceride (mmol/l), HDL-C (mmol/l), LDL-C (mmol/l), glucose (mmol/l) and free fatty acids (FFA) (mmol/l) were measured using an enzymatic in vitro assay (Roche Diagnostics, Mannheim, Germany). The intra- and interassay coefficients of variation (CV) were <2% and <3% for triglycerides, <1% and <2% for HDL-C, <1% and <2% for LDL-C, <2% and <2% for glucose and <2% and <3% for FFA. Serum insulin (pmol/l) and serum cortisol (nmol/l) levels were measured using a chemi-luminescence-based immunoassay (Immulite 2000, Siemens DPC, Los Angeles, CA, USA). Intra- and interassay CV were <6% and <7% for insulin and <7% and <15% for cortisol. Homeostatic model assessment (HOMA), that quantifies insulin resistance and beta cell function, was calculated [29]. The HOMA authors used data from physiological studies to develop mathematical equations describing glucose regulation as a feedback loop. The authors have tested HOMA extensively against other measures of insulin resistance (or its reciprocal, insulin sensitivity) and β-cell function [30], [31], [32]. Modification of Diet in Renal Disease (MDRD) study equation was calculated as a measurement of glomerul filtration rate [33], [34].

### Statistics

Statistical analyses were performed with the Statistical Package for Social Sciences (SPSS 17.0, Chicago, IL, USA). P-values <0.05 (two-tailed) were considered statistically significant. Independent Sample T-tests were used to compare results in survivors and controls and in subgroups. Metabolic syndrome and its separate components was evaluated with Chi-squared tests, all subjects were included. When assessing the metabolic syndrome, information can be lost as outcomes are dichotomized. Metabolic syndrome is designed for use in clinical settings but for research purposes the use of continuous variables provides more information. Therefore multiple linear regression analyses were performed with the outcome parameters glucose, triglycerides, HDL-C, waist circumference, systolic and diastolic blood pressure (components of metabolic syndrome) and insulin, HOMA, LDL-C, FFA (parameters considered associated with metabolic syndrome) as dependent variables. Glucose, insulin, HOMA and triglycerides levels were normally distributed after log-transformation and were expressed in percentages. Logistic regression analysis was performed with the outcome parameter metabolic syndrome (yes/no) as dependent variable and ordinal regression analysis was performed with the number of components of metabolic syndrome as dependent variable. For multiple linear regression analyses subjects treated for diabetes were excluded when evaluating glucose, insulin and FFA; subjects treated for hypertension were excluded when evaluating blood pressure; subjects treated for dyslipidemia were excluded when evaluating triglycerides, HDL-C and LDL-C. All models were corrected for age and sex. When the P-value was <0.200 for the possible confounders educational level, BMI, smoking and physical activity, these confounders were kept in the subsequent models. In model 1 the effects of the diagnoses (dummy variables) were evaluated. In model 2–4 the effects of chemotherapy, nephrectomy, adrenalectomy and abdominal radiotherapy (dummy variables) were evaluated. Additionally, the effects of renal function (as measured by MDRD) and adrenal function (as measured by basal cortisol) were evaluated in model 2. Controls were indicated as not treated for treatment variables. We included SDS for total percentage fat ≥2 as a surrogate adiposity component for metabolic syndrome. Frequency of SDS ≥2 for total percentage fat was compared between survivors treated with and without abdominal radiotherapy.

## Results

### Survivors and Controls

In this study we included 164 subjects, i.e. 103 adult long-term survivors of nephroblastoma and neuroblastoma and 61 control subjects. Out of 88 adult long-term survivors of nephroblastoma who were alive and living in the Netherlands, 67 (28 females) participated (six were lost to follow-up, two were not able to visit the outpatient clinic at the appointed time and 13 refused). Median age was 30.2 (range 18.8–50.8) years and median follow-up time was 26.2 (6.4–48.9) years. Survivors of neuroblastoma stage 4 s who had not received any previous treatment were excluded from this study. Out of 55 remaining neuroblastoma survivors, 36 (21 females) participated (five were lost to follow-up, three were pregnant at the time of the study and six refused). Median age was 29.6 (20.4–46.2) years and median follow-up time was 27.8 (15.0–44.4) years. Survivors that did not participate were not different from participating survivors with respect to baseline characteristics (data not shown).

Fifty-eight percent of survivors had a sibling or friend willing to participate, resulting in 61 participating control subjects (28 females). Thirty-seven control subjects were siblings and 24 were partners/friends. Main reason for control-subjects not to participate was that they had to take a day off work. Baseline and treatment characteristics are shown in Table 1.

### Insulin Resistance

High fasting glucose (or treatment) was present in 14% of controls, 22% of nephroblastoma and 20% of neuroblastoma survivors (Table 2). After adjusting for confounders, glucose levels were lower after adrenalectomy (β = −8.7%, P = 0.001). Adding MDRD (P = 0.364) and basal cortisol (P = 0.038) to this model did not change this resuls (glucose after adrenalectomy β = −9.6, P<0.001) (Table 3). Five survivors were treated for type 2 diabetes at time of the study. Of these five survivors, four received irradiation to the total pancreas and one received irradiation to part of the pancreas. Survivors who received radiotherapy to the total pancreas had higher glucose levels than controls (ß = 10.5, P = 0.002). Insulin levels were not determined by diagnosis, type of chemotherapy, surgery or radiotherapy field (Table 3), even after adjusting for MDRD and basal cortisol.

**Table 2:** Prevalence of components of the metabolic syndrome in nephroblastoma and neuroblastoma survivors compared with controls.

	Controls	Nephroblastoma	P-value^1^	Neuroblastoma	P-value^2^
	(N = 61)	(N = 67)		(N = 36)	
**Fasting glucose** *	14%	22%	0.27	20%	0.49
**Hypertension** *	14%	39%	**0.002**	29%	0.081
**Low HDL-C**	20%	24%	0.66	29%	0.39
**High Triglycerides** *	11%	27%	**0.031**	18%	0.35
**High LDL**	21%	31%	0.20	31%	0.32
***High Waist*** **	*10%*	*6%*	*0.44*	*12%*	*0.73*
**High % total body fat**	n.a.	15%	n.a.	19%	n.a.

* Or treatment.

** Waist circumference is imprecise in irradiated survivors.

^1^Nephroblastoma survivors compared with controls.

**^2^**Neuroblastoma survivors compared with controls (Chi-squared test). All subjects are included. Frequency of fasting glucose does not include one survivor with diabetes type 1.

**Table 3:** Influence of treatment components and diagnosis on parameters.

		Glucose (%)^1^		Triglycerides (%)^2^		HDL-C (mmol/l)^3^		LDL-C (mmol/l)^2^		Insulin (%)^4^		FFA (mmol/l)^5^	
Model	N	152*		149**		154**		153**		150*		151*	
		β	P	β	P	β	P	β	P	β	P	β	P
**1**	**Controls** (N = 61)	*ref*	*ref*	*ref*	*ref*	*ref*	*ref*	*ref*	*ref*	*ref*	*ref*	*ref*	*ref*
	**Nephroblastoma** (N = 67)	−2.9	0.24	30.2	**0.001**	−0.05	0.36	0.16	0.22	22.4	0.132	0.02	0.53
	**Neuroblastoma** (N = 36)	−1.7	0.55	12.9	0.24	−0.10	0.105	0.44	**0.004**	−8.1	0.60	0.05	0.29
**2**	**Controls** (N = 61)	*ref*	*ref*	*ref*	*ref*	*ref*	*ref*	*ref*	*ref*	*ref*	*ref*	*ref*	*ref*
	**Chemotherapy** (N = 90)	1.8	0.55	**28.3**	**0.009**	−0.12	0.054	**0.45**	**0.003**	27.6	0.139	0.05	0.28
	**Nephrectomy** (N = 74)	5.3	0.47	−2.3	0.83	0.07	0.27	−**0.40**	**0.014**	18.8	0.33	−**0.13**	**0.005**
	**Adrenalectomy** (N = 49)	−**8.7**	**0.001**	−10.6	0.24	−0.06	0.31	0.00	1.00	−20.1	0.152	0.04	0.32
	**Rx abdomen** (N = 42)	2.3	0.108	**33.8**	**0.005**	0.02	0.71	**0.32**	**0.049**	−10.6	0.53	**0.16**	**<0.001**
**3**	**Controls** (N = 61)	*ref*	*ref*	*ref*	*ref*	*ref*	*ref*	*ref*	*ref*	*ref*	*ref*	*ref*	*ref*
	**Pancreas – part** (N = 15)	3.9	0.28	19.7	0.159	0.09	0.23	0.46	**0.018**	−3.7	0.85	0.09	0.121
	**Pancreas – total** (N = 27)	**10.5**	**0.002**	**44.8**	**0.002**	−0.07	0.30	0.22	0.23	20.1	0.30	**0.23**	**<0.001**
**4**	**Controls** (N = 61)	*ref*	*ref*	*ref*	*ref*	*ref*	*ref*	*ref*	*ref*	*ref*	*ref*	*ref*	*ref*
	**Liver – part** (N = 19)	**9.5**	**0.013**	**39.1**	**0.015**	−0.09	0.28	0.30	0.151	16.9	0.46	**0.17**	**0.002**
	**Liver – total** (N = 23)	6.3	0.056	**28.4**	**0.030**	0.05	0.45	**0.35**	**0.044**	5.0	0.78	**0.16**	**0.001**

* subjects with treatment for diabetes excluded.

** subjects with treatment for dyslipidemia excluded.

^1^Corrected for age, sex, BMI, physical activity.

^2^Corrected for age, sex, BMI, smoking, physical activity.

3 Corrected for age, sex, BMI, smoking.

4 corrected for age, sex, SES, BMI.

5 corrected for age, sex, physical activity. Glucose, insulin, HOMA and triglycerides levels were normally distributed after log-transformation and were expressed in percentages. ref = reference value (control subjects).

Multiple linear regression analyses were performed using the following strategy: Model 1: the effects of both diagnoses (dummy variables) were added. Model 2: the effects of chemotherapy, nephrectomy, adrenalectomy, abdominal radiotherapy (dummy variables) were added. Additional linear regression analyses were performed according to the following strategy: Model 3: the effects of radiotherapy to the total pancreas and on part of the pancreas (Figure 1) (dummy variables) were added. Model 4: the effect of radiotherapy on the total liver and on part of the liver (Figure 1) (dummy variables) were added. Model 3 and 4 were additionally corrected for the treatment components that were significant in Model 2. P-values indicate the significance of the difference with control subject.

After adjusting for age, sex, educational level, smoking and BMI, HOMA was not significantly different between non-diabetic survivors of nephroblastoma (ß = 16.4%, P = 0.075) and neuroblastoma (ß−4.0%, P = 0.70) and controls. HOMA was not affected by nephrectomy, adrenalectomy or chemotherapy. Basal cortisol was significantly associated with HOMA (P = 0.015), but MDRD was not (P = 0.087). Adding these confounders to Model 2 did not affect results for nephrectomy, adrenalectomy or chemotherapy. Survivors who received radiotherapy to the total pancreas area had higher HOMA levels than controls (ß = 27.0%, P = 0.034), in contrast to survivors who received radiotherapy to part of the pancreas (ß = 3.7%, P = 0.78).

### Blood Pressure

Thirty-nine percent of nephroblastoma survivors had arterial hypertension or treatment for hypertension versus 29% of neuroblastoma survivors and 14% of controls (Table 2). After adjusting for age, sex, BMI and smoking, both nephroblastoma and neuroblastoma survivors had higher systolic (β = 5.6, P = 0.007 and β = 7.1, P = 0.003, respectively) and diastolic (β = 4.8, P = 0.001 and β = 6.0, P = 0.001, respectively) blood pressure levels than controls. Abdominal radiotherapy was associated with higher systolic (β = 6.1, P = 0.021) and diastolic (β = 4.9, P = 0.011) blood pressure. After adding MDRD and basal cortisol to the model abdominal radiotherapy was still associated with systolic (β = 6.2, P = 0.019) and with diastolic (β = 4.8, P = 0.011) blood pressure. Chemotherapy only was associated with higher systolic blood pressure (β = 4.9, P = 0.041), also after adding MDRD and basal cortisol to this model (β = 5.2, P = 0.040).

### Dyslipidemia

High triglyceride levels or treatment for dyslipidemia was registered in 27% of nephroblastoma survivors, 18% of neuroblastoma survivors and 11% in controls (Table 2). After adjusting for confounders, nephroblastoma survivors had higher triglyceride levels (β = 30.2, P = 0.001), which seemed to be determined by abdominal radiotherapy (β = 33.8, P = 0.005) and chemotherapy only (β = 28.3, P = 0.009) (Table 3). Basal cortisol was significantly associated with triglycerides (P<0.001) and MDRD was not (P = 0.225), however this did not change the results (abdominal radiotherapy β = 36.1, P = 0.001) and chemotherapy only β = 27.9, P = 0.007). Survivors who received radiotherapy to the total pancreas had significantly higher triglyceride levels (β = 44.8%, P = 0.002) than controls, in contrast to survivors who received radiotherapy to part of the pancreas (β = 19.7%, P = 0.159) (Table 3). Diagnosis, treatment components and radiotherapy field did not affect the HDL levels (Table 3), which remained the same after adjusting for MDRD and basal cortisol. After adjusting for confounders, LDL-C levels were higher in neuroblastoma survivors (β = 0.44, P = 0.004). Chemotherapy only (β = 0.45, P = 0.003) and abdominal radiotherapy (β = 0.32, P = 0.049) were positively associated with LDL-C levels, whereas nephrectomy was negatively associated with LDL-C levels (β−0.40, P = 0.014). Basal cortisol was significantly associated with LDL-C (P = 0.003) and MDRD was not (P = 0.851), however this did only slightly change these results (chemotherapy only β = 0.48, P = 0.002), abdominal radiotherapy β = 0.31, P = 0.050, nephrectomy β = 0.42, P = 0.011). Survivors who received radiotherapy to the total liver had higher LDL-C levels (β = 0.35, P = 0.044) than controls (Table 3).

After adjusting for confounders, abdominal radiotherapy was positively associated with higher FFA levels (β = 0.16, P<0.001), also after correction for MDRD and basal cortisol (β = 0.17, P<0.001) whereas nephrectomy was negatively associated with FFA levels (β = −0.13, P = 0.005), also after correction for MDRD and basal cortisol (β = 0.14, P = 0.003).

Survivors who received radiotherapy to the total pancreas had significantly higher FFA levels than controls (β = 0.23, P<0.001), in contrast to survivors who received radiotherapy to only part of the pancreas (β = 0.09, P = 0.121) (Table 3).

### Adiposity Measurements

Baseline data regarding weight, height and BMI at diagnosis were available in 84 out of 103 survivors. Median BMI at diagnosis in nephroblastoma survivors was −0.52 (interquartile range (−1.21–0.13) and in neuroblastoma survivors −0.58 (interquartile range −1.99–0.16). After correction for age and sex, nephroblastoma survivors had a smaller waist circumference (β = −4.7 cm, P<0.001) than controls. After additional correction for BMI, low waist circumference was associated with abdominal radiotherapy (β = −5.6, P<0.001) but not with diagnosis or surgery. This effect remained the same after adjusting for MDRD and basal cortisol. It is conceivable that this abnormal waist circumference is determined by anatomical derangements due to abdominal irradiation. Therefore, suggorate markers for adiposity were included in the analyses. After adjusting for age, sex and BMI, abdominal radiotherapy was associated with a lower waist/hip ratio (ß−0.05, P<0.001) and with a lower abdominal fat/tot body fat ratio (ß = −1.9, P<0.001) but not with total body fat percentage (ß = 1.4, P = 0.29).

High waist circumference was present in 14% of the survivors treated without abdominal radiotherapy and in 2% of the survivors treated with abdominal radiotherapy (P = 0.020). Using total body fat (as measured by DXA-scan) as a surrogate marker for adiposity, 9% of the survivors treated without abdominal radiotherapy was categorized as adipose versus 28% of the survivors treated with abdominal radiotherapy (P = 0.018).

### Metabolic Syndrome

**Figure 2:**
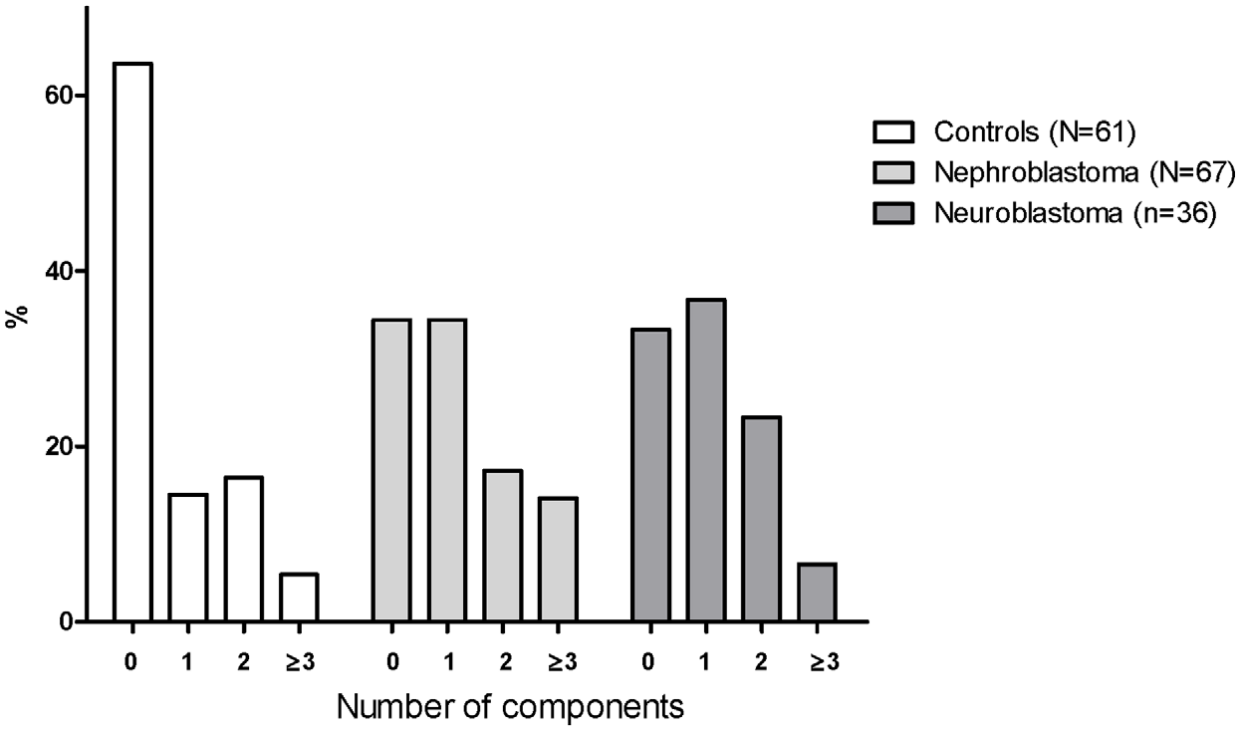
Components of the metabolic syndrome in nephroblastoma and neuroblastoma survivors. Frequency of metabolic syndrome determined according to the definition of the NCEP. Each group in total (0, 1, 2, ≥3 components) equals 100%.

Prevalence of metabolic syndrome according to the NCEP criteria was not significantly different between nephroblastoma and neuroblastoma survivors and controls (Figure 2). After adjusting for age, sex, educational level and BMI, odds ratios (OR) for the frequency of metabolic syndrome as compared with controls were still not significant (OR for nephroblastoma survivors 4.3, P = 0.093, OR for neuroblastoma survivors 2.7, P = 0.38). However, OR for the number of components of the metabolic syndrome were 5.2 (P<0.001) in nephroblastoma and 6.5 in neuroblastoma (P<0.001) survivors.

**Figure 3:**
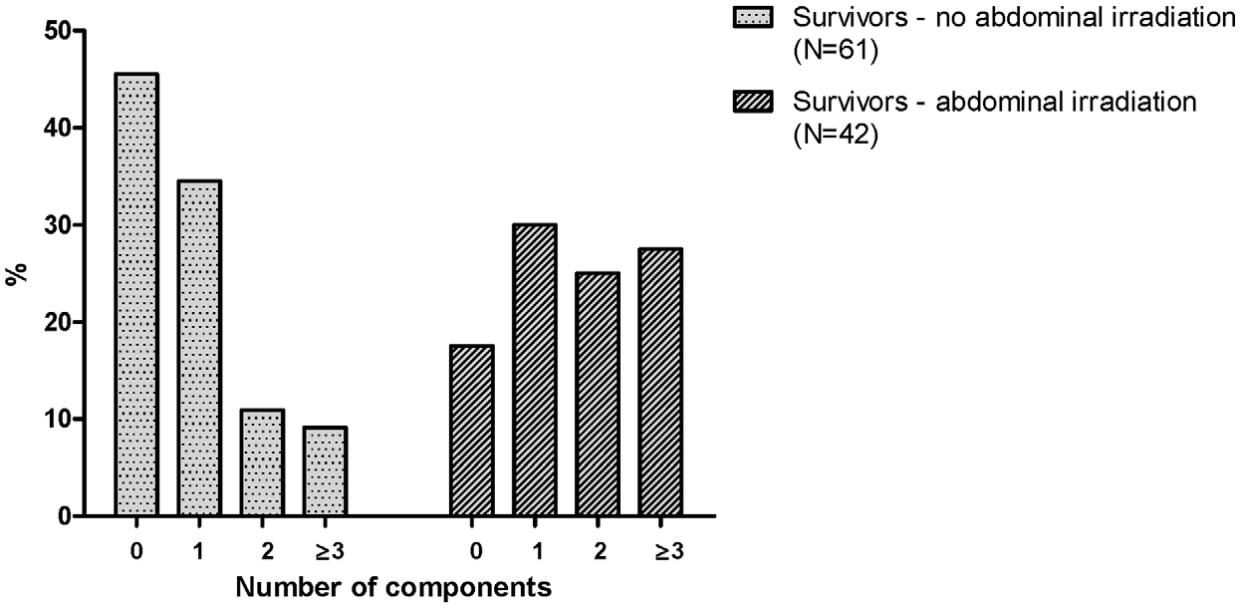
Components of the metabolic syndrome in survivors treated with and without abdominal irradiation. Frequency of the metabolic syndrome determined according to the definition of NCEP but instead of waist circumference, total percentage fat was used as an alternative marker for adiposity. Prevalence of metabolic syndrome (≥3 components) in abdominally irradiated survivors is significantly higher than in controls (P = 0.018) (Chi-squared test). Each group in total (0, 1, 2, ≥3 components) equals 100%.

Subsequently, total fat percentage as measured by DXA scan was used as a surrogate marker for the adiposity component of metabolic syndrome. Metabolic syndrome was almost three times more frequent in abdominally irradiated survivors (28%) than in non-irradiated survivors (9%, P = 0.018) (Figure 3).

## Discussion

In the present study we show that abdominal irradiation is the main determinant of metabolic syndrome in long-term survivors of nephroblastoma and neuroblastoma, which was mainly due to hypertension, adiposity and dyslipidemia. Metabolic syndrome occurred three times more often in abdominally irradiated survivors, which is significant, especially when the relatively young age of the survivors and the fact that the incidence of cardiovascular diseases increases with age is taken into account [35].

In the current study, hypertension and dyslipidemia in nephroblastoma and neuroblastoma survivors were mainly determined by previously administered abdominal irradiation. This is consistent with findings of the Childhood Cancer Survivor Study as we described in the introduction of this manuscript. We hypothesize that the effect of abdominal radiotherapy on development of (components of the) metabolic syndrome may be due to radiation induced damage on individual organs. Therefore, we categorized patients according to administered radiation field. Although HOMA and insulin levels were not different from controls in the non-diabetic survivors, we show that radiotherapy involving both head and tail of the pancreas influenced the occurrence of dyslipidemia and insulin resistance significantly. The pancreas has always been considered as relatively insensitive to radiation [36], [37], however similar to the recent report of de Vathaire et al. [38], our findings indicate that the pancreas is an organ at risk during radiation therapy. This probably results from radiation induced apoptosis of pancreatic beta cells, and consequently to decreased insulin production, the thereby induced hyperglycemia, elevated FFA levels and hypertriglyceridemia and insulin resistance. We also show that abdominal irradiation involving the complete liver influences LDL-C levels, in contrast to irradiation involving only part of the liver. These results indicate that non-irradiated liver and non-irradiated pancreas fields may have the capacity to compensate for impaired liver and pancreas function of the irradiated fields. Although we were only able to categorize radiation fields, these single centre study results emphasize the need for studies in larger cohorts of childhood cancer survivors involving detailed radiation dosimetry, as this might help to unravel the mechanism behind the development of metabolic syndrome.

This is the first study that acknowledges the need for surrogate markers for adiposity in survivors after abdominal irradiation with a deformed abdominal area. We show that the measurement of total body fat probably provides a more reliable measurement of adiposity than waist circumference in abdominally irradiated survivors. It should be considered that abdominal fat is a better predictor of visceral fat than total body fat, however this needs the use of a computerised tomography (CT) or Magnetic Resonance Imaging (MRI) scan, which is expensive, time-consuming and undesirable in childhood cancer survivors who have often already been exposed to teratogenic treatments. Clinicians and researchers however need to be aware of the underestimation of adiposity and metabolic syndrome and, subsequently, the risk for cardiovascular diseases, when using waist circumference as a parameter in this specific subgroup.

Besides abdominal radiotherapy, we observed chemotherapy only to be associated with higher systolic blood pressure and dyslipidemia. Although the exact mechanism remains to be elucidated, it might well be that chemotherapeutic agents damage the vascular endothelium, resulting in vascular alterations.

Furthermore, we found nephrectomy to be associated with lower LDL-C and FFA levels, whereas adrenalectomy was associated with lower glucose levels. Both types of surgery thus do not seem to increase the risk for development of metabolic syndrome. We additionally adjusted for glomerular filtration rate and for basal cortisol to be able to analyze the direct effect renal and adrenal function on the outcome parameters. Similar to what we previously showed, basal cortisol levels were associated with insulin resistance and dyslipidemia [39]. We found no effect of glomerular filtration rate on our outcome parameters. This indicates that the effect of nephrectomy on lower FFA and LDL-C levels, is not determined by decreased renal function. In our study, abdominal ultrasounds performed as part of clinical follow-up did not show structural defects in the survivors. In future studies, imaging modalities might however provide extra information, although it needs to be considered that size of kidney or adrenal gland is not necessarily related to function. Specific studies evaluating the correlation between imaging modalities and function of organs however are of interest and might identify practical tools for the future.

Although our study was limited by small sample sizes due to therapy subgroups, we are the first to report on metabolic syndrome and associated measures in survivors of nephroblastoma and neuroblastoma. To be able to increase power, we recommend accrual of higher number of control subjects for future studies. Furthermore, to evaluate the effects of baseline characteristics on the outcome variables, we performed a subanalysis and found BMI at diagnosis not to be associated with BMI at follow-up, or with waist circumference, diastolic blood pressure, triglycerides and HDL-C. This indicates that components of the metabolic syndrome in childhood cancer survivors are determined by treatment factors and less by baseline patients characteristics. Higher BMI at diagnosis was associated with lower blood pressure and lower glucose levels at follow-up, which indicates that the results we described in our paper might be underestimated, but are unlikely to be overestimated.

In conclusion, our study demonstrated that nephroblastoma and neuroblastoma survivors are at increased risk for developing (components of) metabolic syndrome, and that abdominal radiotherapy is the main determinant of its occurrence. Our findings indicate that the pancreas and the liver are organs at risk for damage due to radiation therapy. Consequently, when planning treatment, a radiation dose as low as possible including the smallest field, should be administered. This is especially applicable for children treated for neuroblastoma and nephroblastoma. Surrogate markers for adiposity, such as total body fat, should be included in future studies in order to get insight in the true frequency of metabolic syndrome after abdominal irradiation. Prospective studies in large cohorts including radiation dosimetry are necessary to be able to unravel the underlying mechanisms behind the increased risk for developing components of metabolic syndrome after abdominal radiotherapy.

## References

[1] Bernstein ML, Leclerc JM, Bunin G, Brisson L, Robison L, et al (1992) A population-based study of neuroblastoma incidence, survival, and mortality in North America. J Clin Oncol 10: 323–329. 1732433 10.1200/JCO.1992.10.2.323

[2] Bernstein L, Linet M, Smith M, Olshan AF (1999) Cancer incidence and survival among children and adolescents: United States SEER Program 1975–1995, SEER Program. National Cancer Institute 1999: p.79–90.

[3] van Waas M, Neggers SJ, van der Lelij AJ, Pieters R, van den Heuvel-Eibrink MM (2010) The metabolic syndrome in adult survivors of childhood cancer, a review. J Pediatr Hematol Oncol 32: 171–179. 20186100 10.1097/MPH.0b013e3181d419c3

[4] van Waas M, Neggers SJ, Pieters R, van den Heuvel-Eibrink MM (2010) Components of the metabolic syndrome in 500 adult long-term survivors of childhood cancer. Ann Oncol 21: 1121–1126. 19850641 10.1093/annonc/mdp414

[5] van Waas M, Neggers SJ, Te Winkel ML, Beishuizen A, Pieters R, et al.. (2011) Endocrine late sequelae in long-term survivors of childhood non-Hodgkin lymphoma. Ann Oncol. 10.1093/annonc/mdr511 22048153

[6] van Beek RD, van den Heuvel-Eibrink MM, Hakvoort-Cammel FG, van den Bos C, van der Pal HJ, et al (2009) Bone mineral density, growth, and thyroid function in long-term survivors of pediatric Hodgkin’s lymphoma treated with chemotherapy only. J Clin Endocrinol Metab 94: 1904–1909. 19293271 10.1210/jc.2008-0622

[7] Blijdorp K, van den Heuvel-Eibrink M, Pieters R, Boot A, Sluimer J, et al.. (2011) The limited screening value of insulin-like growth factor-i as a marker for alterations in body composition in very long-term adult survivors of childhood cancer. Pediatr Blood Cancer. 10.1002/pbc.24015 22162176

[8] Meacham LR, Chow EJ, Ness KK, Kamdar KY, Chen Y, et al (2010) Cardiovascular risk factors in adult survivors of pediatric cancer–a report from the childhood cancer survivor study. Cancer Epidemiol Biomarkers Prev 19: 170–181. 20056636 10.1158/1055-9965.EPI-09-0555 PMC2805162

[9] Geenen MM, Bakker PJ, Kremer LC, Kastelein JJ, van Leeuwen FE (2010) Increased prevalence of risk factors for cardiovascular disease in long-term survivors of acute lymphoblastic leukemia and Wilms tumor treated with radiotherapy. Pediatr Blood Cancer 55: 690–697. 20589650 10.1002/pbc.22518

[10] van Dijk IW, Oldenburger F, Cardous-Ubbink MC, Geenen MM, Heinen RC, et al (2010) Evaluation of late adverse events in long-term wilms’ tumor survivors. Int J Radiat Oncol Biol Phys 78: 370–378. 20137867 10.1016/j.ijrobp.2009.08.016

[11] Grundy SM, Becker D, Clark LT, Cooper RS, Denke MA, et al (2001) Executive Summary of The Third Report of The National Cholesterol Education Program (NCEP) Expert Panel on Detection, Evaluation, And Treatment of High Blood Cholesterol In Adults (Adult Treatment Panel III). Jama 285: 2486–2497. 11368702 10.1001/jama.285.19.2486

[12] Reaven GM (1988) Banting lecture 1988. Role of insulin resistance in human disease. Diabetes 37: 1595–1607. 3056758 10.2337/diab.37.12.1595

[13] Kershaw EE, Flier JS (2004) Adipose tissue as an endocrine organ. J Clin Endocrinol Metab 89: 2548–2556. 15181022 10.1210/jc.2004-0395

[14] Yusuf S, Hawken S, Ounpuu S, Dans T, Avezum A, et al (2004) Effect of potentially modifiable risk factors associated with myocardial infarction in 52 countries (the INTERHEART study): case-control study. Lancet 364: 937–952. 15364185 10.1016/S0140-6736(04)17018-9

[15] Montague CT, O’Rahilly S (2000) The perils of portliness: causes and consequences of visceral adiposity. Diabetes 49: 883–888. 10866038 10.2337/diabetes.49.6.883

[16] Kern PA, Ranganathan S, Li C, Wood L, Ranganathan G (2001) Adipose tissue tumor necrosis factor and interleukin-6 expression in human obesity and insulin resistance. Am J Physiol Endocrinol Metab 280: E745–751. 11287357 10.1152/ajpendo.2001.280.5.E745

[17] Marette A (2003) Molecular mechanisms of inflammation in obesity-linked insulin resistance. Int J Obes Relat Metab Disord 27 Suppl 3S46–48. 14704744 10.1038/sj.ijo.0802500

[18] Laverdiere C, Cheung NK, Kushner BH, Kramer K, Modak S, et al (2005) Long-term complications in survivors of advanced stage neuroblastoma. Pediatr Blood Cancer 45: 324–332. 15714447 10.1002/pbc.20331

[19] Sasso G, Greco N, Murino P, Sasso FS (2010) Late toxicity in Wilms tumor patients treated with radiotherapy at 15 years of median follow-up. J Pediatr Hematol Oncol 32: e264–267. 20736847 10.1097/MPH.0b013e3181e7931a

[20] Wright KD, Green DM, Daw NC (2009) Late effects of treatment for wilms tumor. Pediatr Hematol Oncol 26: 407–413. 19657990 10.1080/08880010903019344 PMC2829307

[21] Wendel-Vos GC, Schuit AJ, Saris WH, Kromhout D (2003) Reproducibility and relative validity of the short questionnaire to assess health-enhancing physical activity. J Clin Epidemiol 56: 1163–1169. 14680666 10.1016/s0895-4356(03)00220-8

[22] Khosla T, Lowe CR (1967) Indices of obesity derived from body weight and height. Br J Prev Soc Med 21: 122–128. 6033482 10.1136/jech.21.3.122 PMC1059084

[23] Blokstra A SH, Mesquita HBBd, Seidell JC, Verschuren WMM (1997) Monitoring van Risicofactoren en Gezondheid in Nederland (MORGEN-project), 1993–1997. Leefstijl- en risicofactoren: prevalenties en trends. Available: http://wwwrivmnl/bibliotheek/rapporten/263200008pdf. Accessed 2009 Aug 3.

[24] Fredriks AM, van Buuren S, Burgmeijer RJ, Meulmeester JF, Beuker RJ, et al (2000) Continuing positive secular growth change in The Netherlands 1955–1997. Pediatr Res 47: 316–323. 10709729 10.1203/00006450-200003000-00006

[25] WHO (2008) Waist circumference and Waist-Hip Ratio: Report of a WHO Expert Consultation. Geneva, 8–11 December 2008.

[26] Expert Panel on Detection E, Treatment of High Blood Cholesterol in A (2001) Executive Summary of The Third Report of The National Cholesterol Education Program (NCEP) Expert Panel on Detection, Evaluation, And Treatment of High Blood Cholesterol In Adults (Adult Treatment Panel III). JAMA 285: 2486–2497. 11368702 10.1001/jama.285.19.2486

[27] Pimenta N, Santa-Clara H, Fragoso IJ (2010) Comparison of body composition and body fat distribution of patients following a cardiac rehabilitation program and sedentary patients. Rev Port Cardiol 29: 1163–1180. 21066969

[28] van der Sluis IM, de Ridder MA, Boot AM, Krenning EP, de Muinck Keizer-Schrama SM (2002) Reference data for bone density and body composition measured with dual energy x ray absorptiometry in white children and young adults. Arch Dis Child 87: 341–347; discussion 341–347. 10.1136/adc.87.4.341 PMC1763043 12244017

[29] Matthews DR, Hosker JP, Rudenski AS, Naylor BA, Treacher DF, et al (1985) Homeostasis model assessment: insulin resistance and beta-cell function from fasting plasma glucose and insulin concentrations in man. Diabetologia 28: 412–419. 3899825 10.1007/BF00280883

[30] Wallace TM, Levy JC, Matthews DR (2004) Use and abuse of HOMA modeling. Diabetes Care 27: 1487–1495. 15161807 10.2337/diacare.27.6.1487

[31] Hermans MP, Levy JC, Morris RJ, Turner RC (1999) Comparison of tests of beta-cell function across a range of glucose tolerance from normal to diabetes. Diabetes 48: 1779–1786. 10480608 10.2337/diabetes.48.9.1779

[32] Hermans MP, Levy JC, Morris RJ, Turner RC (1999) Comparison of insulin sensitivity tests across a range of glucose tolerance from normal to diabetes. Diabetologia 42: 678–687. 10382587 10.1007/s001250051215

[33] Levey AS, Eckardt KU, Tsukamoto Y, Levin A, Coresh J, et al (2005) Definition and classification of chronic kidney disease: a position statement from Kidney Disease: Improving Global Outcomes (KDIGO). Kidney Int 67: 2089–2100. 15882252 10.1111/j.1523-1755.2005.00365.x

[34] National Kidney (2002) Foundation (2002) K/DOQI clinical practice guidelines for chronic kidney disease: evaluation, classification, and stratification. Am J Kidney Dis 39: S1–266. 11904577

[35] Mackay J, Mensah G (2004) The Atlas of Heart Disease and Stroke. World Health Organization.

[36] Radiation CotBEoI (1990) Health Effects of Exposure to Low Levels of Ionizing Radiation. BEIR V Washington, DC: National Academy Press. 25032334

[37] Shimizu Y, Kato H, Schull WJ (1990) Studies of the mortality of A-bomb survivors. 9. Mortality, 1950–1985: Part 2. Cancer mortality based on the recently revised doses (DS86). Radiat Res 121: 120–141. 2305030

[38] de Vathaire F, El-Fayech C, Ben Ayed FF, Haddy N, Guibout C, et al. Radiation dose to the pancreas and risk of diabetes mellitus in childhood cancer survivors: a retrospective cohort study. Lancet Oncol. 10.1016/S1470-2045(12)70323-6 22921663

[39] Van Waas M, Pieters R, van Eck JP, Van Noesel MM, Van der Lelij AJ, et al.. (2012) Adrenal function in adult long-term survivors of nephroblastoma and neuroblastoma. European Journal of Cancer, epub ahead of print. 10.1016/j.ejca.2012.02.046 22513228

